# Self-Organization Leads to Supraoptimal Performance in Public Transportation Systems

**DOI:** 10.1371/journal.pone.0021469

**Published:** 2011-06-30

**Authors:** Carlos Gershenson

**Affiliations:** 1 Departamento de Ciencias de la Computación, Instituto de Investigaciones en Matemáticas Aplicadas y en Sistemas, Universidad Nacional Autónoma de México, México, Distrito Federal, México; 2 Centro de Ciencias de la Complejidad, Universidad Nacional Autónoma de México, México, Distrito Federal, México; University of Zaragoza, Spain

## Abstract

The performance of public transportation systems affects a large part of the population. Current theory assumes that passengers are served optimally when vehicles arrive at stations with regular intervals. In this paper, it is shown that self-organization can improve the performance of public transportation systems beyond the theoretical optimum by responding adaptively to local conditions. This is possible because of a “slower-is-faster” effect, where passengers wait more time at stations but total travel times are reduced. The proposed self-organizing method uses “antipheromones” to regulate headways, which are inspired by the stigmergy (communication via environment) of some ant colonies.

## Introduction

Public transportation systems play an essential role in urban mobility. There are more than a hundred million daily users in the 100 busiest metro systems in the world [1]. Considering that more than half of the world's population lives in cities [2], a relevant percentage of citizens is affected by the performance of buses, trams, trains, metros, and other public transportation systems.

**Figure 1:**
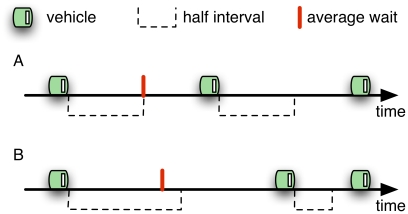
Different headways. A. Equal headways lead to shorter passenger waiting times at stations. B. Unequal headways lead to longer waiting times because there is a higher probability of passenger arrival within longer headways.

Theory indicates that passengers arriving at stations randomly will be optimally served if the headways–the time intervals between vehicles–are equal [3] (See Fig. 1). However, if no restrictions are applied, the configuration of equal headways is always unstable [4]. Different approaches have been used to promote the stability of equal headways [5].

Traditionally, transportation systems are optimized for an expected average demand. However, the precise demand changes constantly–in this case, passengers at stations. Vehicles with equal headways might have to wait unnecessarily at stations to keep regular intervals, increasing travel times. An alternative is to use self-organization to let the system adapt by itself to changes in demand [6].

In the next section, the public transportation system model that we used is presented, along with the proposed self-organizing method. Computer simulation results follow, showing that a configuration of unequal, but adaptive, headways can lead to a supraoptimal performance of public transportation systems. A discussion closes the paper. For a Spanish language version of the abstract, please see Spanish Abstract S1.

## Methods

A multi-agent simulation was used to make qualitative statistical experiments on a previously proposed metro-style model of public transportation systems [4]. Space and time are discrete. Vehicles move along a cyclic track with stations where passengers board and descend. Vehicles travel at a constant speed of one “patch” (discrete spatial unit) per “tick” (discrete temporal unit), unless there is a vehicle in front, the vehicle is at a station where passengers are boarding or descending, or a method restricts the departure from a station. Vehicles and stations occupy one patch in the environment. Passengers arrive at stations randomly, where the time between passenger arrivals is determined with a Poisson distribution of mean 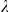. Thus, a lower 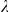 implies less time between passenger arrivals, i.e. a higher passenger inflow. Passengers wait until a vehicle arrives at a station and board after passengers descend, only if the vehicle has not reached its maximum capacity. Passengers travel a random number of stations that is less than the total number of stations, i.e. they do not visit the same station twice, even when the track is cyclic. The waiting time of passengers at stations is considered from the moment a passenger enters the simulation until a vehicle is boarded. The total waiting time of passengers is considered from the moment a passenger enters the simulation until she exits, i.e. the total travel time minus the minimum travel time for the number of stations traveled. The minimum travel time is useful to calculate the theoretical optimum. It is equivalent to the travel time assuming a single passenger in the whole system, with vehicles ready at every station to serve her.

The reader is invited to access the simulation with a Java-enabled browser at the URL: http://turing.iimas.unam.mx/cgg/NetLogo/4.1/metro.html (or http://tinyurl.com/antipheromones for short). The source code is available from the site.

### Model properties

Equal headways are always unstable under no restrictions in this public transportation model. Since passengers arrive randomly with a Poisson distribution, some stations will have more passengers than others. Thus, vehicles will wait more time at stations with a higher passenger demand, and less time at stations with a lower passenger demand. Also, vehicles with more passengers will spend more time at stations waiting for them to exit. Thus, the heterogeneous usage of vehicles leads to heterogeneous travel times. This implies that faster vehicles will catch up slower ones, reducing their headway. Slower vehicles will increase the headway with faster vehicles in front of them. This leads to the formation of “platoons”, where the first vehicle is slowed down by the high demand at stations, caused by long headways, and subsequent vehicles idle behind the slow vehicle.

If a minimum waiting time is imposed for vehicles at stations, equal headways can be maintained for low passenger densities. When there is a high passenger demand, busy stations cause some vehicles to be delayed, breaking the equal headway configuration.

When apart from the minimum waiting time a maximum waiting time at stations is imposed, equal headways can be maintained when the minimum and maximum waiting times are equal, i.e. all vehicles remain at stations for an equal amount of time. However, this implies that some passengers might not be allowed to board into busy vehicles. Nevertheless, this allows an even load distribution among vehicles, leading to a higher efficiency of the system.

The best minimum and maximum waiting times at stations depend on the passenger demand. This led to the proposal of an adaptive method (*MX*) where the maximum waiting time is adjusted depending on the total number of passengers in the system [4].

Fixing the waiting times at stations of vehicles is a *necessary but not sufficient* condition for equal headways. For example, if more passengers are exiting a vehicle at a station than the minimum waiting time, then the vehicle will be delayed, as each passenger takes one tick to exit the vehicle. Also, *MX* can maintain equal headways, but not recover them. In other words, if initial conditions have unequal headways, these will be maintained with *MX*. This was the main motivation for exploring a new, self-organizing method: to achieve regular headways starting from non-homogeneous conditions. The supraoptimal performance was an unexpected consequence of the method.

### Self-organizing method

A self-organizing method (*SO*) was devised to regulate adaptively the behavior of vehicles depending on the current state of the system, exploiting only local information. The method was inspired in the stigmergy (communication via environment) of social insects [7], [8]. Some ant species leave pheromone trails that evaporate with time. The pheromone intensity is used as a signal to coordinate the behavior of a colony. The proposed method uses the concept of “antipheromone”, where the environment regularly increases the concentration of antipheromone and vehicles remove the antipheromone as they travel. Like this, the antipheromone concentration informs a vehicle of the headway to the vehicle in front. The method keeps headways regular–but not equal–with a margin that depends on the number passengers at the current station.

Antipheromone concentration is increased regularly (one unit per tick) in every patch and erased when a vehicle leaves a patch. This behavior is opposite to traditional pheromones, which would have a high concentration after a vehicle left a patch, and “evaporate” with time. In other words, pheromone concentration is reduced with time, while antipheromone concentration is increased.

A headway regulation method could vary speeds of vehicles to promote equal headways. Still, the self-organizing method manages to regulate efficiently a public transportation system by only restricting or forcing the departure of vehicles at stations. These measures can counteract the two causes of headway instability: vehicles going faster than expected and vehicles going slower than expected (see Discussion below). Only one of them is not enough, e.g. only delaying vehicles at stations would imply that all vehicles would go at the speed of the slowest one, giving a performance similar to the case without restrictions, although maintaining equal headways.

**Figure 2:**
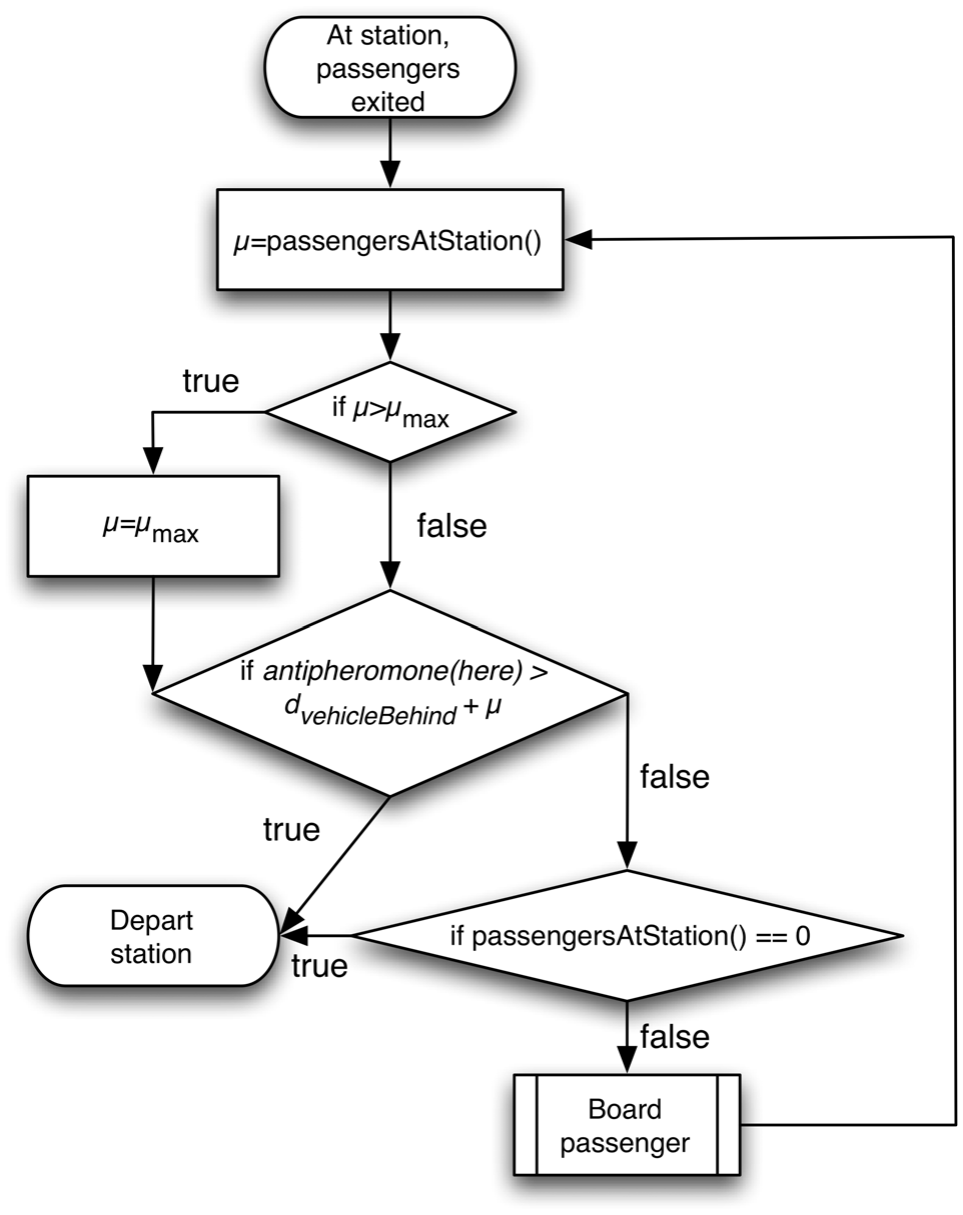
Flow diagram of self-organizing method (*SO *).

A flow diagram of the self-organizing algorithm is shown in Fig. 2: Vehicles arrive at stations and let passengers exit. Then, the number of passengers at the station is counted in parameter 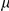, which will be used as a margin. If 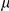 is greater than a maximum margin 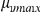, 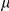 is bounded to 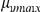. Afterwards, the antipheromone concentration–which represents the *time* since the last vehicle left the station–is compared with the *distance* to the vehicle behind, taking into consideration the margin 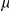 which reflects how many passengers are waiting at the station. The distance is used because it is uncertain how long the vehicle behind will take to reach the current station. Since space and time are abstract, speed (1 patch/tick), distances (patches), and passenger boarding times (1 tick per passenger) are comparable. Thus, normalization is not required. If the antipheromone value is higher than the distance plus 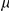, then the vehicle departs, even if there are passengers waiting to board. This is because a high antipheromone concentration implies that there is a long headway with the vehicle ahead. This decision is flexible depending on how many passengers are waiting at the station, represented by 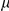. If 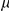 is high, then the antipheromone value should be higher to trigger the departure of the vehicle, i.e. the headway with the vehicle in front is increased. If the antipheromone concentration is still low, i.e. a vehicle recently left the station, the algorithm considers whether there are any passengers at the station waiting to board. If there are none, the vehicle departs to prevent idling. Otherwise, the passengers are allowed to board, and the algorithm starts again its evaluation.

Vehicles follow the self-organizing algorithm independently, i.e. there is no direct communication between vehicles, nor a central control. Since the behavior of the system is determined by the local interactions of vehicles, it is useful to describe the system as self-organizing [9]. Notice that waiting times at stations are dynamic and decentralized, depending on the local demand at each station.

## Results

In the following experiments, five vehicles and five stations were used, with a maximum vehicle capacity of fifty passengers. The cyclic track had a length of 121 patches. Variations of these parameters were explored and they did not affect the qualitative outcome of the experiments. Each simulation run is initialized with empty stations. Then, the simulation runs for 5000 initial ticks. Data is averaged for the subsequent 5000 ticks. For each method and each 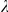 value 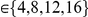, one hundred simulation runs were performed and used to produce the boxplots shown below.

The self-organizing method (*SO*) was compared with a default method (*DF*)–where headways are always unstable–and a previously proposed method (*MX*) that maintains equal headways by adapting the maximum waiting times of vehicles at stations depending on the total number of passengers in the system [4]. In a first set of experiments, a regular scenario was used, with the same passenger demand (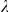) at each station, equidistant stations and equidistant initial positions of vehicles. Even when this is not common in real systems, it is the most favorable scenario for equal headways. Self-organization offers greater improvements on irregular, more realistic scenarios.

**Figure 3:**
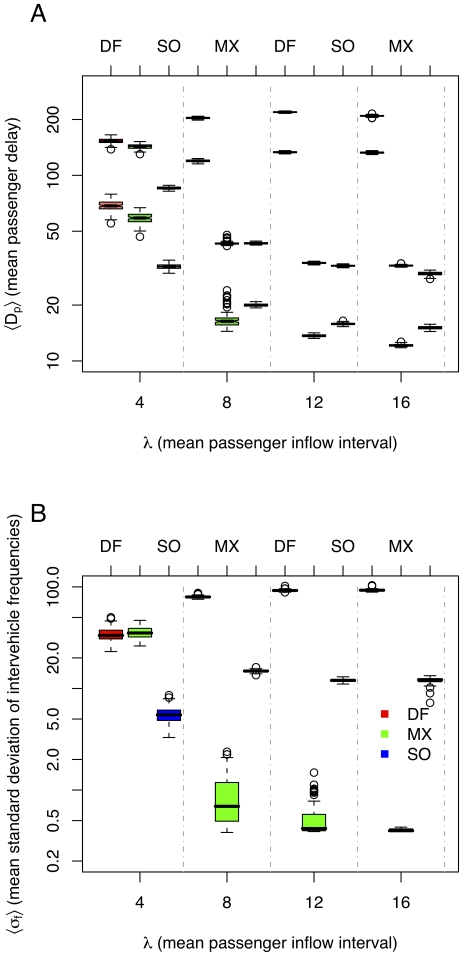
Results for homogeneous scenario. A. Passenger delays for methods: “default” (*DF*), “max” (*MX*), and “self-organizing” (*SO*), for different passenger demands (lower 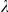 means higher demand). Lower boxes at each column show waiting times at stations. Higher boxes show total waiting times. B. Headway standard deviations. Lower 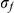 implies more regular headways. *DF* shows unstable headways, *MX* equal headways (except for 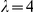), and *SO* adaptive headways. Notice logarithmic scale.

Simulation results for the homogeneous scenario are shown in Fig. 3. It can be seen that *DF* gives a poor performance, always leading to unstable headways. *MX* gives the lowest waiting times at stations while maintaining equal headways. This is consistent with theory [3]. *SO* has higher waiting times at stations, but the lowest total waiting times.

**Figure 4:**
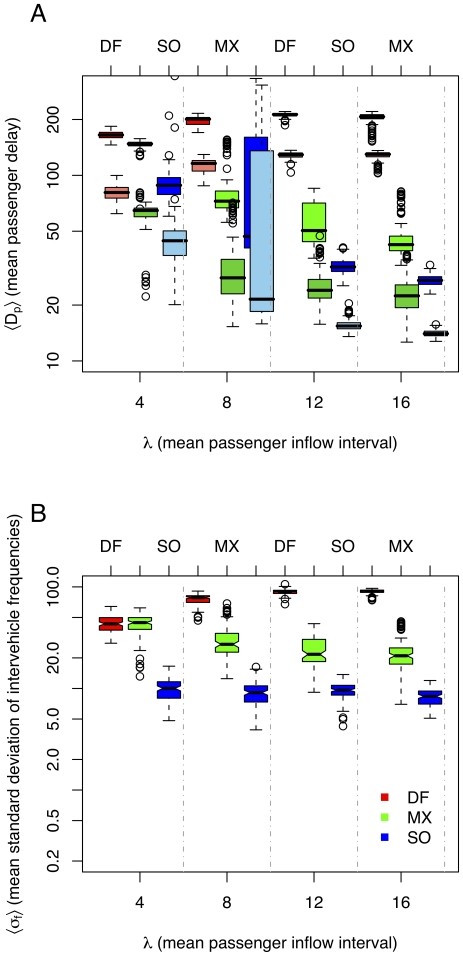
Results for non-homogeneous scenario. A. Passenger delays for methods: “default” (*DF*), “max” (*MX*), and “self-organizing” (*SO*), for different passenger inflow intervals 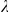. Lower boxes, slightly shifted to the right, at each column show waiting times at stations. Higher boxes show total waiting times. B. Headway standard deviations. Lower 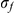 implies more regular headways. Notice logarithmic scale.

Fig. 4 shows results for another set of experiments preformed on a non-homogeneous scenario: stations are placed randomly in the simulation keeping a minimum interstation distance of five “patches” (see Methods). Each station 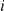 has a different 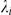 to determine its passenger inflow. The values of 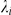 are selected randomly during initialization of each simulation run from a Poisson distribution with a mean 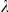. This leads to a greater standard deviations in the results, since the passenger demand and interstation distances can vary from run to run depending on the random initialization. Vehicles are initialized with random positions, instead of equidistant.

The results shown in Fig. 4 indicate that the performance difference in considerably increased for this more realistic scenario. *DF* delivers a poor performance without any restriction at stations, with similar waiting times to the homogeneous scenario. Since there is a random initial position of vehicles, *MX* maintains this vehicular configuration, i.e. without equal headways. This is because *MX* can maintain equal headways, but cannot recover them once they are lost. *SO* obtains a performance similar to the homogeneous scenario and is able to maintain adaptive headways.

In a similar heterogeneous scenario, but with equidistant initialization of vehicles, *MX* manages to maintain equal headways, and waiting times at stations are lower for *MX* than for *SO*. Still, the total waiting times for passengers are considerably lower for *SO*.

Since the self-organizing method can lead to regular–although not equal–headways, starting from random initial conditions, it can be concluded that the self-organizing method is *sufficient* to maintain regular headways, leading to a supraoptimal system performance.

All of the previous experiments were performed in a metro-style scenario, i.e. with no interaction with traffic lights (as it is the case for bus rapid transit systems) or other types of vehicular traffic (as it is the case for bus lines without dedicated lanes). The option to generate a number of traffic lights was implemented in the simulation. Further experiments showed that *DF* and *MX* are highly sensitive to the positions and periods of the traffic lights. Small changes in these parameters lead to large performance differences. Moreover, the best values for these parameters change with passenger density. *SO* adapts to a majority of scenarios, although some combinations of parameters also affect negatively its performance. An integration of a public transportation system with self-organizing traffic lights [10], [11] would solve this problem.

## Discussion

In previous work, it was shown that an equal headway configuration is always unstable if there are no restrictions on passengers or vehicles [4], as it is the case with *DF*. This is because passengers arriving randomly at stations will cause different waiting and travel times for vehicles, leading to unstable headways. There are two general causes of headway instability: a) vehicles going faster than expected, and b) vehicles going slower than expected. It was shown that both causes have to be taken into consideration to maintain equal headways [4]. For the first cause, vehicles going faster than expected, vehicle idling is enough for instability prevention. For the second cause, vehicles going slower than expected, less obvious measures must be taken. In the presented abstract scenario, vehicles are delayed because they serve more passengers. The solution for reducing their delay is to prevent passengers from boarding these vehicles. Even when some passengers will wait more at stations, maintaining regular headways will ensure that their total waiting and travel times are less than with *DF*, where passenger boarding is not restricted.

Results presented in the previous section show that public transportation systems can be improved beyond the optimum of current theory, which focuses on waiting times at stations, assuming that travel times are independent of the boarding policies. However, equal headways can lead to slower travel times due to potential idling at stations. This is because of a “slower-is-faster” effect [12], [13], where passengers may wait more time to board a vehicle at stations, but vehicles wait less time at each station. The total waiting times are less than in the equal headway configuration. The self-organizing method reduces total waiting times–in spite of increasing waiting times at stations–by relaxing the equal headway restriction but without leading to headway instability. This solution is not predefined, it is responsive to the local conditions of each station and vehicle. This enables *SO* to adapt at much faster timescales than *MX*, leading to supraoptimal performance.

When the conditions of a system are changing at a particular temporal scale, the controller for that system must adapt at that same temporal scale to obtain the best results [14]–[16]. In the case of public transportation systems, changes occur at the seconds scale, since different configurations of vehicles and passengers require different responses from the controller. The proposed self-organizing method matches this temporal scale with the aid of antipheromones and the current number of passengers at stations.

The technical requirements for implementing the self-organizing method are available: antipheromones can be implemented with timers at stations that vehicles reset as they depart. Sensors to measure the number of passengers at stations exist, as well as devices to count the number of boarding and exiting passengers. Also, a good estimate of passengers can be obtained with mobile phone proximity sensors, since most people carry a mobile phone. The distance between vehicles can be also obtained with sensors or GPS.

The social aspect of implementing the algorithm is more complicated, since passengers sometimes are restricted from boarding a vehicle. Purposeful architecture, station, and vehicle configurations, user education, a clear explanation of the benefits to the public and timely information can contribute to its adoption.

## Supporting Information

Spanish Abstract S1 Spanish language version of the abstract.
(PDF)
