# Supplementary material for: Self-Organization Leads to Supraoptimal Performance in Public Transportation Systems
Source: PLoS One. 2011 Jun 30;6(6):e21469. doi: 10.1371/journal.pone.0021469 (PMC3127858; doi:10.1371/journal.pone.0021469)
Supplement: Spanish Abstract S1 — Spanish language version of the abstract. (PDF) [file pone.0021469.s001.pdf]

Self-organization leads to supraoptimal performance in public transportation systems

Carlos Gershenson[1,2,*]
1 Departamento de Ciencias de la Computación, Instituto de Investigaciones en Matemáticas Aplicadas y en Sistemas, Universidad Nacional Autónoma de México, México, D.F., México.
2 Centro de Ciencias de la Complejidad, Universidad Nacional Autónoma de México, México, D.F., México.
∗ **E-mail: cgg@unam.mx**

Supporting Information

Spanish Abstract S1

El desempeño de los sistemas de transporte público afecta a una parte considerable de la población. La teoría actual asume que los pasajeros son servidos de manera óptima cuando los vehículos llegan a las estaciones a intervalos regulares. En este trabajo se muestra que la auto-organización puede mejorar el desempeño de los sistemas de transporte público más allá del óptimo teórico al responder de manera adaptativa a condiciones locales. Esto es posible por un efecto "lento-es-más-rápido", donde los pasajeros esperan más tiempo en las estaciones pero los tiempos de viaje totales son reducidos. El método auto-organizante propuesto usa "antiferomonas" para regular intervalos, las cuales están inspiradas en la estigmergía (comunicación a través del medio ambiente) de algunas colonias de hormigas.
